# Measurement tools for the diagnosis of nasal septal deviation: a systematic review

**DOI:** 10.1186/1916-0216-43-11

**Published:** 2014-04-24

**Authors:** Tehnia Aziz, Vincent L Biron, Kal Ansari, Carlos Flores-Mir

**Affiliations:** 1 University of Alberta, Faculty of Medicine and Dentistry, School of Dentistry, Edmonton, Alberta, Canada; 2 Department of Surgery, Division of Otolaryngology-Head and Neck Surgery, University of Alberta, Edmonton, Alberta, Canada

## Abstract

**Objective:**

To perform a systematic review of measurement tools utilized for the diagnosis of nasal septal deviation (NSD).

**Methods:**

Electronic database searches were performed using MEDLINE (from 1966 to second week of August 2013), EMBASE (from 1966 to second week of August 2013), Web of Science (from 1945 to second week of August 2013) and all Evidence Based Medicine Reviews Files (EBMR); Cochrane Database of Systematic Review (CDSR), Cochrane Central Register of Controlled Trials (CCTR), Cochrane Methodology Register (CMR), Database of Abstracts of Reviews of Effects (DARE), American College of Physicians Journal Club (ACP Journal Club), Health Technology Assessments (HTA), NHS Economic Evaluation Database (NHSEED) till the second quarter of 2013. The search terms used in database searches were ‘nasal septum’, ‘deviation’, ‘diagnosis’, ‘nose deformities’ and ‘nose malformation’. The studies were reviewed using the updated Quality Assessment of Diagnostic Accuracy Studies (QUADAS-2) tool.

**Results:**

Online searches resulted in 23 abstracts after removal of duplicates that resulted from overlap of studies between the electronic databases. An additional 15 abstracts were excluded due to lack of relevance. A total of 8 studies were systematically reviewed.

**Conclusions:**

Diagnostic modalities such as acoustic rhinometry, rhinomanometry and nasal spectral sound analysis may be useful in identifying NSD in anterior region of the nasal cavity, but these tests in isolation are of limited utility. Compared to anterior rhinoscopy, nasal endoscopy, and imaging the above mentioned index tests lack sensitivity and specificity in identifying the presence, location, and severity of NSD.

## Introduction

Nasal septal deviation (NSD) is a common diagnosis made by otolaryngologists but is one that is not usually based on objective measurements. As a result, there can be a significant inter-observer variability in terms of diagnosing the condition, verifying its precise location, quantifying the degree of deviation, and assessing its clinical impact on patients. This subjectivity can lead to unnecessary surgical treatments, patient complications and low patient satisfaction rates. In the current era of evidence-based medicine, society demands that surgical interventions demonstrate clinically significant improvements. Since there is no consensus agreement about diagnosing NSD objectively, interventions treating NSD lack a strong evidence base. Interventions not supported by evidence-based medicine are at risk of being curtailed by publicly funded healthcare systems.

The nasal septum is a midline support structure of the nasal cavity. Aside from being a key support mechanism of the nose and a major determinant of its shape, the space between the septum and lateral walls of the nasal cavity regulates nasal airflow and respiration. Within the nasal cavity, a straight septum enables laminar airflow, allowing the inspired air to be warmed, cleaned and humidified and thus optimized for gas exchange. Conversely, a deviated nasal septum can contribute to various degrees of nasal obstruction and altered nasal respiration [1,2].

Deviation of the nasal septum is a common structural cause of nasal obstruction and can arise from dislocation of the quandriangular cartilage from its bony boundaries, or from an intrinsic deformity affecting the vomer, perpendicular plate of ethmoid and/or the quadrilateral cartilage itself [3]. In neonates, prevalence of septal deviation can vary from 1.45% [4] to 6.3% [5]. A recent study [6] analyzed the prevalence of septal deviations in newborns and found that it can be as high as 22% in children delivered vaginally, while birth from a caesarean section resulted in only 4% NSD. Trauma to the septum from vaginal birth was suggested to be a common cause of NSD. The prevalence of NSD in school-aged children aged 6-15 years was roughly 20% when assessed on occipitomental projection radiographs, while a positive clinical diagnosis was made in approximately 10% of the same cohort of children [7].

Overall, the etiology of NSD can be classified as congenital, genetic effects causing aberrant growth, trauma [8], infection, or even mass effect from nasal cavity neoplasms [9]. A recent study suggested that a long sphenoid process of the septal cartilage could also contribute to NSD [10].

Depending on the severity and location of NSD in adults, it can lead to mouth breathing, nasal crusting, epistaxis, and sinusitis [11]. In infants, severe and bilateral NSD can result in poor feeding/and or choking from food in the respiratory tract [6]. Dental findings of patients with nasal obstruction resulting from NSD have been reported as Class 2 malocclusion with increased anterior facial height, retrognathic maxilla and mandible with increased overjet and constricted transverse maxillary dimension [12].

The wide range of reported incidences of NSD mentioned above is largely due to a lack of standardized objective criteria for making the diagnosis of NSD. However, other mitigating factors such as presence of turbinate hypertrophy, rhinitis, nasal valve collapse, nasal cycle and the complexity of the three dimensional geometry of the nasal cavity make the diagnosis even more challenging. Essentially, there seems to be no acceptable protocol for establishing the diagnosis of NSD. Diagnostic tests namely acoustic rhinometry (AR), rhinomanometry (RMM) and nasal spectral sound analysis (NSSA) have been documented in the literature to assess septal deviation. Acoustic rhinometry (AR) assesses nasal patency based on the measurement of acoustic reflection of a sound signal in the nose by structures within the nasal cavity [13]. Rhinomanometry provides a dynamic physiologic assessment of the nose by measuring transnasal pressure and nasal volume airflow to calculate nasal resistance [13]. Nasal sound spectral analysis (NSSA) can provide an indirect method of dynamically assessing nasal airflow by analyzing noise in the nasal cavity caused by turbulent nasal airflow [14].

The purpose of this systematic review is to investigate the diagnostic modalities utilized to assess NSD. To our knowledge, no such review has been conducted, and considering the clinical manifestations and consequences of NSD, it would be beneficial to have an evidence-based diagnostic schema for NSD.

## Methods

An electronic database search was conducted with the assistance of a senior librarian specializing in health sciences database searches. The electronic databases were MEDLINE (from 1966 to second week of August 2013), EMBASE (from 1966 to second week of August 2013), Web of Science (from 1945 to second week of August 2013) and all Evidence Based Medicine Reviews Files (EBMR); Cochrane Database of Systematic Review (CDSR), Cochrane Central Register of Controlled Trials (CCTR), Cochrane Methodology Register (CMR), Database of Abstracts of Reviews of Effects (DARE), American College of Physicians Journal Club (ACP Journal Club), Health Technology Assessments (HTA), NHS Economic Evaluation Database (NHSEED) until the second quarter of 2013. The search terms used in database searches were ‘nasal septum’, ‘deviation’, ‘diagnosis’, ‘nose deformities’ and ‘nose malformation’ (Additional file 1). The following inclusion criteria were used to initially select studies from the abstracts and titles located through electronic database search.

Inclusion criteria consisted the following: human studies only, no case reports or conference proceedings, abstracts that discussed diagnosis of nasal obstruction with reference to septal deviation and no neonatal studies. Since the diagnosis and etiology of septal deviation in neonates is considered a separate entity it was not included in this systematic review.

Two authors (T.A. and K.A.) independently reviewed the title and abstracts of the database searches. Full text of all studies that appeared to meet the inclusion criteria were retrieved along with ones that had insufficient information in the abstracts to make a final decision regarding their inclusion. The references of retrieved articles were also manually searched for additional studies that could be included in the systematic review. The authors (T.A and K.A.) independently assessed full articles obtained for inclusion in the systematic review and any disagreement was settled through discussion until a consensus was reached.

The following exclusion criteria were finally applied to the studies after retrieval of full text of articles: Any concurrent sino-nasal pathology in patients that would preclude diagnosis of nasal septal deviation was excluded, examples of such conditions included, but not limited to, were septal perforation, chronic rhinitis, chonal atresia, enlarged turbinates, nasal polyps etc; computer simulations of airflow to mimic septal deviation were not included, as these were not in vivo studies.; studies including patients with prior septal surgery were not included, as this would reduce the detection rate of diagnosing nasal septal deviation; patients that did not receive any topical nasal decongestant prior to administering the diagnostic test were not included in this study. Minimizing mucosal swelling of septum will reduce the false positive rates of detecting nasal septal deviation.

Methodological scoring to assess quality of included studies was performed through use of the updated Quality Assessment of Diagnostic Accuracy Studies (QUADAS-2) tool (changed reference# 28 to QUADAS-2) [15]. It was established that the quality assessment would be through analysis of individual components and not the overall quality score.

## Results

**Figure 1:**
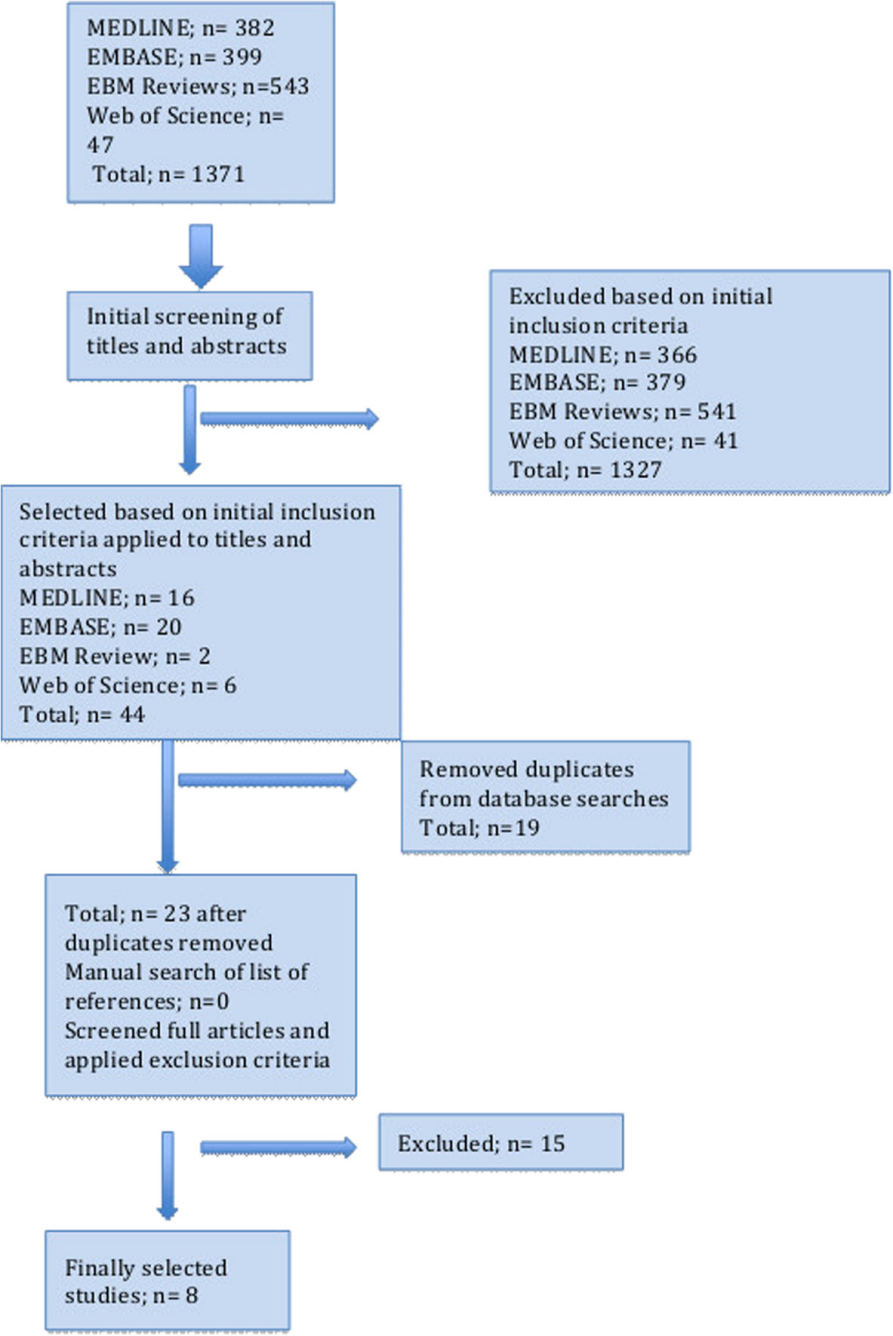
Summary of systematic review process.

**Figure 2:**
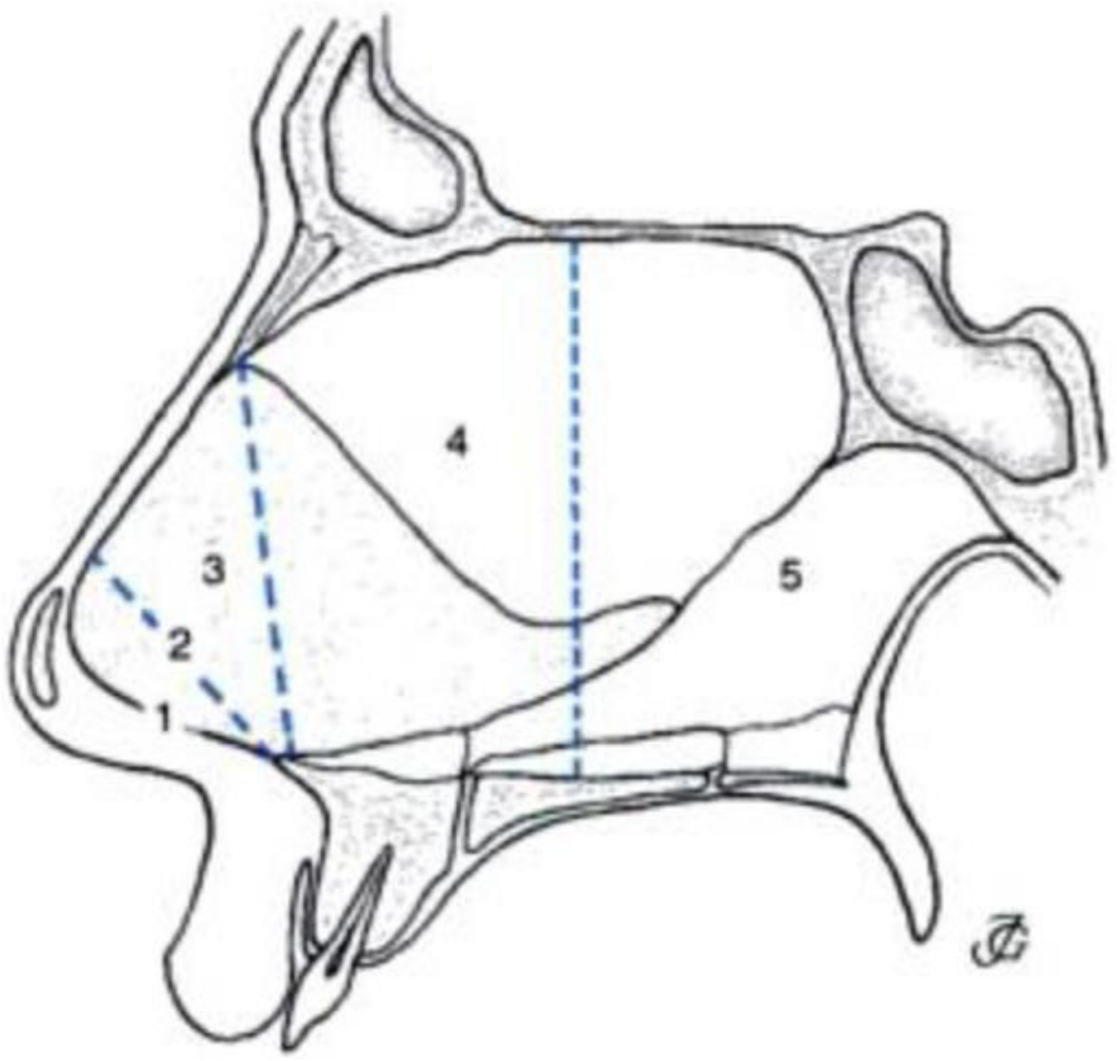
**Areas of the nasal cavity according to Cottle.** Area 1: nostril. Area 2:nasal valve. Area 3: area underneath the bony and cartilaginous vault, also called the attic. Area 4: anterior aspect of the nasal cavity including the heads of the turbinates and the infundibulum. Area 5: the posterior aspect of the nasal cavity, including the tails of the turbinates. (Adapted from Egbert H et al. Incorrect terminology in nasal anatomy and surgery, suggestions for improvement. Rhinology, 2003; 41:129-133).

The flow chart of the electronic database search and final selection of studies to be included in the systematic review is outlined (Figure 1). Online searches resulted in 23 abstracts [13-36] after removal of duplicates that resulted from overlap of studies between the electronic databases. Fifteen studies were excluded [16-30] after full review of the articles and reasons for their exclusion are listed in Table 1. This resulted in a total of 8 studies [13,14,31-36] to be included in this systematic review. Key details of the included studies are listed in Table 2. Three studies [14,32,33] discussed the analysis of nasal sound intensity on expiration [32], inspiration [14] and both inspiration/expiration [33] in 2000-4000 Hz frequency interval as diagnostic modality for nasal septal deviation. It was suggested in two of these studies that there was a positive correlation between severity of NSD and in intensity of nasal sounds [14,32]. Three other articles [13,31,35] concluded that acoustic rhinometry (AR) was a reliable tool in diagnosing anterio-caudal NSDs (Figure 2) [13,35]. One study [31] concluded that the sensitivity of AR in detecting anterio-caudal septal deviations is 57% and specificity is 70% when assessing even minor septal deviations that are visible on CT scans, but might not be clinically relevant. Another study [35] concluded that acoustic rhinometry could detect NSD due to statistically significant differences in the cross sectional areas and nasal cavity volumes between obstructed and unobstructed sides of the nose. One article on rhinomanometry concluded that it has limited diagnostic value in the clinical setting due to its ability to only diagnose major septal deviations in the anterior region and these were found only in a minority of the sample patients [34]. Finally, one study [36] concluded that physical examination from nasal endoscopy/anterior rhinoscopy is an accurate method of diagnosing septal deviation patients requiring septal surgery.

**Table 1:** Studies excluded from our systematic review

**Study**	**Reason for exclusion**
Cho GS et al [16]	Discussed association between subjective sensation of nasal obstruction with respect to different locations in the nose with lack of reference to diagnosis of nasal septal deviation
Liu T et al [17]	Computer simulations of nasal airflow in nasal obstruction/septal deviation
Chen XB at al [18]	Computer simulations of nasal airflow in nasal obstruction/septal deviation
Hanif J et al [19]	Little reference to diagnosis of septal deviation, discussed quantification of severity of nasal septum for future surgery
Filho DI et al [20]	Little or no reference to diagnosis of nasal septal deviation
Cole P et al [21]	Computer simulations of nasal airflow in nasal obstruction/septal deviation
Farhadi, M [22]	Unclear on inclusion of patients with only septal deviation/nasal obstruction from other causes
Kahveci OK [23]	Only addressed efficacy of NOSE scale in patients receiving septal surgery
Rujanavej V et al [24]	Diagnosis of septal deviation made with concurrent nasal obstruction and sinonasal disease
Gogniashvilli G et al [25]	Prevalence study of physiological/pathological septal deviation
Garcia GJ et al [26]	Computer simulations of nasal airflow in nasal obstruction/septal deviation
Pirila T et al [27]	Discussed patient satisfaction with septoplasty, without reference to diagnosis of septal deviation
Chandra RK et al [28]	Review of nasal obstruction
Benninger MS [29]	Excluded patients with nasal septal deviation
Cuddihy PJ et al [30]	Almost half of the sample of patients had concurrent rhinitis

**Table 2:** Summary of Studies Included in our Systematic Review

**Study**	**Study group**	**Control group**	**Diagnostic measure (s)**	**Sensitivity (SN)/Specificity (SP)**	**Results**
Choi et al [14]	43 patients Ages 18 to 48 years (mean 35 +/-13 yrs)	n = 40 Ages 20 to 50 years (mean 32+/-24 years)	NSSA compared with PNIF and VAS	SN = 86% and SP = 83% for NSSA in septal deviation patients at 2000-4000 Hz interval. SN = 79% and SP = 78% for PNIF	Correlation between PNIF and NSSA for frequency interval 2000- 4000 Hz in deviated patients (r = 0.72, p < 0.01)
Mamikoglu et al [31]	24 patients Ages 14 to 67 (median 36)	No control group	AR compared with CT scans MCA measured 2, 4 and 6 cm from the nostril	SN of AR in detecting anterior septal deviations is 57% and SP is 70% when assessing minor septal deviations seen on CT	AR and CT correlate well at if deviation present at a distance of 2 cm from anterior nose (r = 0.73, p < 0.001). Correlation decreases past 4 cm and AR is not accurate beyond 6 cm
Tahamiler et al [32]	61 patients Ages 18 to 66 years (mean 32 +/-11)	No control group	Comparison between AR and VAS using OR at 200- 6000 Hz (MCA 1 measured 2.2 cm from anterior nose)	Not mentioned	Weak correlation but significant results for OR at 2000-4000 Hz and 4000-6000 Hz interval (r = 0.5, p < 0.01) with AR for 2.2 cm from the vestibule for measurement taken ipsilateral to the deviation. Between VAS and OR at 2000-4000 Hz (r = 0.41, p < 0.01) for ipsilateral deviation
Tahamiler et al [33]	n = 68, Ages 18 to 54 years, (mean 32)	n = 61 Ages 17 to 56 years, (mean 34)	Expiratory/inspiratory nasal sound with OR, Compared with VAS and RMM	None mentioned	OR correlates well with VAS/RMM and can be useful tool is measuring nasal patency in 2000-4000 Hz interval (p < 0.0001)
Huygen et al [34]	n = 193, no ages given. (Site of septal deviation; vestibule, valve, anterior-superior portion/central and posterior areas)	n = 33, 21-67 years of age	RMM (mean flow at transnasal pressure of 150 Pa) vs rhinoscopic measurement of deviation	None mentioned	RMM is a poor tool for localization of deviation.
					Had 80% detection rate for only severe deviations in nasal vestibule and valve
Szucs et al [13]	n = 50 Ages 18 to 64, (mean 33) Group 1, n = 8 severe septal deviation anterior nasal cavity up to 2.5 cm from columella, Cottle area I and II Group 2, n = 14 moderate deviation, anterior nasal cavity Cottle area I and II Group 3, n = 12, middle nasal cavity between 2.5 to 4.5 cm from columella, Cottle area IV Group 4, n = 16, posterior nasal cavity, between 4.5 to 8 cm from columella Cottle area V (Figure 2)	n = 15	RMM and AR. Inspiratory and expiratory nasal airway resistance (NAR) at 75 and 150 Pa measured for RMM. MCA and volume of nasal cavity at deviation measured by AR	Both AR and RMM show sensitivity in diagnosis of severe and moderate septal deviation in the anterior part of nasal cavity. Not sensitive enough in middle/ posterior deviations	p <0.05 for MCA, Volume and NAR at 75 and 150 Pa for anterior septal deviation. p > 0.05 for MCA, Volume, and NAR at and 150 Pa for middle and posterior deviations
Huang et al [35]	n = 77 (significant septal deviation); Ages 19-74 yrs, mean age = 39	n = 89 Ages 19-74 yrs, mean age = 39	AR; Mean MCA (anterior 1-5 cm from the anterior nose) Total V (between points at the nostril to 5 cm into the nose)	No sensitivity values given but concluded AR is a sensitive tool to determine structural abnormality	mMCA (p = 0.001) and Total V (p = 0.04) measured on the narrower side was smaller than in the wider part of nasal cavity indicating volume compensation
Sedaghat et al [36]	n = 137 74 males, 63 females mean age = 42 years All had septal deviation	No control group	Nasal endoscopy, anterior rhinoscopy, physical exam	SN = 86.9% and SP = 91.8%	PPV = 93.6% and NPV = 96.4% for septal surgery. Clinical assessment of patients with deviated nasal septum is accurate in predicting them needing medical intervention

*AR* Acoustic Rhinometry, *CT* computed tomography, *MCA* Minimal cross sectional area (mMCA: mean minimal cross sectional area, average of right and left nostrils), *NAR* nasal airway resistance *NSSA* nasal sound spectral analysis, *NPV* negative predictive value, *OR* Odiosoft-Rhino, *PNIF* peak nasal inspiratory flow, *PPV* positive predictive value, *RMM* rhinomanometry, *V* Total Volume (average of right and left nostrils), *VAS* Visual analogue score.

Results from QUADAS-2 tool are listed in Table 3. Most studies selected patients that were representative of the ones receiving the test in a clinical setting and clearly described selection criteria (low risk of bias and lack of applicability concerns for patient selection domain). Most of them described execution of index test to enable replication (high applicability of index test domain). However, none except one study [13] identified and explained patient withdrawal (high risk of bias for flow and timing domain). In all studies except one [36], index tests were performed with the knowledge of the reference tests (high risk of bias for index test).

**Table 3:** Methodological assessment of included studies using the Quality Assessment of Diagnostic Accuracy Studies (QUADAS-2) Checklist

**Study**	**Risk of bias**				**Applicability concerns**		
	**Patient**	**Index**	**Reference**	**Flow and**	**Patient**	**Index test**	**Reference**
	**selection**	**test**	**standard**	**timing**	**selection**		**standard**
Choi et al [14]	LR	HR	LR	HR	U	LR	LR
Mamikoglu et al [31]	LR	HR	LR	HR	U	U	LR
Tahamiler et al [32]	LR	HR	LR	HR	LR	LR	LR
Tahamiler et al [33]	LR	HR	LR	HR	LR	LR	LR
Huygen et al [34]	LR	HR	LR	HR	LR	U	LR
Szucs et al [13]	LR	HR	LR	LR	LR	LR	LR
Huang et al [35]	U	HR	LR	HR	U	LR	LR
Sedaghat et al [36]	LR	LR	U	HR	LR	LR	LR

*LR* = Low risk, *HR* = High risk, *U* = Unclear risk.

## Discussion

Nasal septal deviation (NSD) is a common clinical entity encountered in general otolaryngology-head and neck surgery. Upon review of the literature, no single test was identified as a gold standard of diagnosis of septal deviation. The diagnosis of NSD is generally ascertained after assimilating information gathered from a variety of sources including the patient’s history, physical examination of the nose and anterior rhinoscopy, nasal endoscopy, and imaging [31].

Ideally, surgical interventions should be supported be strong evidence based medicine, with a diagnosis based on objective testing and criteria. Clinical inquiry from patients usually lacks sensitivity and specificity, especially as an isolated diagnostic tool in detecting NSD, possibly due to the presence of numerous co-existing and confounding pathologies. Anterior rhinoscopy and nasal endoscopy performed in the decongested state can diagnose the location and severity of nasal septal deviations, but it is an uncomfortable test that is subject to significant inter-rater variability [1,31]. Imaging studies such as CT scans and MRIs can provide accurate three-dimensional diagnosis of NSD but are typically utilized in the clinical arena to assess paranasal pathology (i.e. sinusitis) rather than isolated NSD [1,31]. As accurate as they can be in diagnosing NSD, the former exposes patients unnecessarily to radiation while both modalities can be expensive [31]. More readily available and less expensive diagnostic modalities have been created to objectively assess the nasal cavity patency. These diagnostic tests included in this systematic review are acoustic rhinometry [13,31,35], rhinomanometry [13,33,34] and nasal sound spectral analysis [29,31,32], all carried out in the decongested state.

Acoustic rhinometry (AR) assesses nasal patency based on the measurement of acoustic reflection of a sound signal in the nose by structures within the nasal cavity. AR analyses the initial and reflected sound waves creating a plot of the cross sectional area of the nasal cavity as a function of the distance from the nasal cavity entrance [13]. Once this data is obtained, nasal volumes can also be calculated using AR. Unlike anterior rhinoscopy and nasal endoscopy, AR provides objective data. Typical minimal cross sectional areas (MCA) are encountered as defined distances from the anterior nasal aperture. In one study [31], they were defined as MCA 1 at 2 cm represents the anterior end of the inferior turbinate and internal nasal valve; MCA 2 at 4 cm represents the anterior part of the middle turbinate; and MCA 3 at 6 cm represents the middle portion of the middle turbinate. This study along with two other [13,35] on acoustic rhinometry concluded that AR becomes less accurate when measurements are made past MCA 1 of the anterior nasal cavity and are completely unreliable past MCA 3. Because MCA 1 in fact represents the internal nasal valve area of the external nose, which is the narrowest part of the nasal passage, it is the most susceptible nasal airflow obstruction in the setting of NSD [37]. Diminished accuracy of AR past the anterior portion of the inferior nasal turbinate (around 2 cm distance from the nostril) could also be due to complicated intranasal anatomy posteriorly that leads to dispersion of acoustic energy [31]. In fact, Mamikoglu et al [31] compared acoustic rhinometry and CT scan in diagnosing NSD, and found a positive correlation between MCA 1 and CT results. In particular, it was determined that the sensitivity of detecting anterior NSD is 54% while the specificity was 70%. Most of these deviations in this study were classified as “mild”. Sensitivity and specificity would have been higher if the study contained a greater proportion of patients with more severe NSDs. However, unlike physical exam and imaging, acoustic rhinometry cannot distinguish DNS from other obstructing nasal pathology.

While AR provides a static view of the nasal cavity, rhinomanometry (RMM) provides a dynamic physiologic assessment of the nose. Based on the laws of fluid dynamics, it quantifies nasal ventilation by measuring transnasal pressure and nasal volume airflow to calculate nasal resistance [13]. Nasal resistance is an internationally accepted index of nasal patency [38]. Huygen *et al*[34] concluded that minor deviations may defy detection by rhinomanometry as the detection rate (22%) of septal deviation was very similar the false positive rate of 24%. Furthermore, they found that RMM was most accurate in identifying larger NSDs in the anterior flow limiting regions of the nose including the nasal vestibule and valve area. Similarly, another study [13] on RMM reported that it is a sensitive tool in identifying septal deviations in anterior part of the nasal cavity, but was unable to determine the location of NSD. Although RMM quantifies the functional impact on nasal flow mechanics caused by these larger anterior based NSD, these anterior NSDs are nevertheless more easily diagnosed by simply performing anterior rhinoscopy. In fact, almost all studies in this systematic review had patients undergo assessment with anterior rhinoscopy and nasal endoscopy to detect severity and location of septal deviation prior to administration of the index test.

In contrast to administering RMM, which can be cumbersome and time consuming [14], nasal sound spectral analysis (NSSA) with Odiosoft-Rhino (OR) can provide an indirect method of dynamically assessing nasal airflow. NSSA analyses noise in the nasal cavity caused by turbulent nasal airflow. It is also easy and inexpensive to conduct [14]. Unlike AR and RMM, NSSA does not require any nasal cannulation, which distorts the nasal cavity, and could skew the measurements [14,33]. In order to accurately quantify this noise, NSSA must be conducted in a quiet room, a minor limitation of this test that is also incidentally experienced with AR. Like AR and RMM, each side of the nasal cavity can be evaluated independently, so side differences can be noted. In essence, one would expect that greater the physical nasal obstruction, greater the turbulent airflow, and louder the noise detected on NSSA testing. One study [14] found a significant difference between nasal inspiratory sound intensity of the NSD patient group and normal controls. The sensitivity and specificity were 86% and 83% respectively in terms of diagnosing isolated NSD. This study [14] also found a correlation between the severity of the deflection and the intensity of the inspiratory nasal sound in the 2000 to 4000 Hz interval. In a cohort with unilateral NSD in another study [32], expiratory sounds at the 2000-4000 Hz and 4000-6000 Hz intervals were found to be significantly louder on the deviated side than the other side of the nose. In same group of patients, Tahamilar *et al*[32] found a positive correlation between visual analog scores assessing the subjective feeling of nasal obstruction and expiratory NSSA measurements and also a direct correlation between the severity of NSD and expiratory NSSA. Furthermore, expiratory NSSA positively correlated with AR findings at MCA 1 region of the nose, that being the internal nasal valve flow limiting segment of the anterior nose. In one study [14] NSSA was compared with peak nasal inspiratory flow (PNIF). PNIF is another measurement of nasal airflow that is obtained with a portable inspiratory flowmeter. This study found a statistically significant lower PNIF values in the NSD group compared to normal controls and a positive correlation with NSSA. According to this paper [14] sensitivity and specificity of PNIF is 79% and 77% respectively for detecting NSDs. However, a limitation of NSSA (and RMM) is that the actual location of the NSD could not be ascertained. A recently published systematic review evaluated the efficacy of septoplasty for treatment of nasal obstruction concluded that AR, RMM and PINF are all valid objective measures to assess nasal patency in patients undergoing surgery [39].

Standardized criteria for assessing the symptom of nasal obstruction caused by NSD can be quantified using validated visual analog scales. However, the results from subjective assessments of nasal obstruction from visual analog scores (VAS) are flawed in patients with chronic DNS who may have simply become accustomed to breathing with limited nasal airflow. This was demonstrated in a study [35] found that only 30 out of 77 patients with significant nasal septal deviation complained subjectively of nasal obstruction. Conversely, out of 89 rhinoscopically normal patients 32 had subjective complaints of nasal obstruction, making VAS for assessing nasal obstruction caused by NSD challenging. There are a number of reasons why there is poor correlation between the subjective sensation of nasal obstruction and objective tests of nasal obstruction; the foremost being is that nasal sensation is relatively poorly understood [30]. Studies included in this systematic review were assessed by QUADAS-2 and several methodological flaws were identified. One major limitation of these diagnostic studies was that anterior rhinoscopy, nasal endoscopy and/or CT scans were conducted to make the diagnosis of NSD prior to the use of diagnostic modalities such as acoustic rhinometry, rhinomanometry and nasal sound analysis (high risk of bias for index test). It was not clear in most studies whether the same examiner conducted all the diagnostic tests. Only one study [33] reported blinding of the examiner for the diagnostic tests conducted. This could lead to review bias [13] whereby interpretation of the results of the diagnostic test such as acoustic rhinometry could be altered by the knowledge of the results from nasal endoscopy and may lead to increased diagnostic accuracy of index tests.

## Conclusions

In summary, diagnostic modalities such as acoustic rhinometry, rhinomanometry and nasal spectral sound analysis may be useful in identifying NSD in anterior region of the nasal cavity, but these tests alone add little value to diagnosis. Compared to anterior rhinoscopy, nasal endoscopy, and imaging the above mentioned index tests lack sensitivity and specificity in identifying the presence, location, and severity of NSD.

## Competing interests

The authors declare that they have no competing interests.

## Authors’ contributions

TA carried out database searches, collected data, performed data analysis and drafted the original manuscript. VB, KA and CF participated in drafting the final manuscript. All authors read and approved the final manuscript.

## Supplementary Material

Additional file 1 Database searches performed in this systematic review. Click here for file
