# Supplementary material for: Measurement tools for the diagnosis of nasal septal deviation: a systematic review
Source: J Otolaryngol Head Neck Surg. 2014 Apr 24;43(1):11. doi: 10.1186/1916-0216-43-11 (PMC4042609; doi:10.1186/1916-0216-43-11)
Supplement: Additional file 1 — Database searches performed in this systematic review. [file 1916-0216-43-11-S1.docx]

**Additional file 1: Database searches performed in this systematic review.**

| MEDLINE/EMBASE and EMBR | Web of Science |
| --- | --- |
| 1. Exp Nasal Septum/ 2. Exp Nose deformities/ 3. Deviat*.mp 4. Exp Diagnosis/ 5. Diagnos*.mp   A. 1 OR 2 AND 3  B. 4 OR 5  Combine A and B | Nasal septal deviation* (Topic)  AND  Diagnosis* (Topic) |
